# Description and distribution of three morphotypes of the *Eucyclopsserrulatus* group (Crustacea, Copepoda, Cyclopoida) from Algerian water bodies

**DOI:** 10.3897/BDJ.11.e100981

**Published:** 2023-03-02

**Authors:** Safia Akli-Bidi

**Affiliations:** 1 Department of Ecology and Environment. Laboratory of Dynamic and Biodiversity. Faculty of Biological Sciences. University of Sciences and Technology Houari Boumediene,. Algiers. Algeria., Bab Ezzouar, Algeria Department of Ecology and Environment. Laboratory of Dynamic and Biodiversity. Faculty of Biological Sciences. University of Sciences and Technology Houari Boumediene,. Algiers. Algeria. Bab Ezzouar Algeria

**Keywords:** distribution, *Eucyclopsserrulatus* group, morphotypes, morphometrics, microcharacters

## Abstract

**Background:**

Examination of *Eucyclops* populations coming from Algerian water bodies and identified as Eucyclopscf.serrulatus showed three morphotypes, based on morphometric characters and microcharacters. Morphotype 1 was the most abundant, collected in the east and the west of Algeria. Morphotype 2 was sampled in the south of the country, characterised by posterolaterally elongated thoracic segments and the fourth thoracic segment bearing cilia on its lateral angles. Morphotype 3 was found in a small temporary pond in the north of the country and was the smallest one. Other differences were observed on surface microcharacters of antenna basipodite, coxopodite and intercoxal plate of the fourth leg. The characters of the most widespread morphotype (morphotype1) were stable in all localities despite the fact that these were located in two geographically separated regions (eastern and western Algeria).

**New information:**

Three morphotypes of *Eucyclopsserrulatus* group (Fisher, 1851) (Crustacea, Copepoda, Cyclopoida) from Algerian water bodies were identified. The characters of the most widespread morphotype (morphotype 1) were stable in all localities despite the fact that these were located in two geographically separated regions (eastern and western Algeria).

## Introduction

The genus *Eucyclops* (Copepoda, Cyclopoida) comprises about one hundred species and 15 subspecies (Dussart and Defaye 2006, Alekseev and Defaye 2011, Mercado-Salas and Suarez-Morales 2014). The last revision of the whole genus was done by Lindberg (1957) for African *Eucyclops*. Several revisions of *Eucyclops* were published: for Australia by Morton (1990), for Ukraine by Monchenko (1974), for Japan by Ishida (2002), for Mexico by Mercado-Salas and Suarez-Morales (2014) and for Palearctic by Alekseev and Defaye (2011) and Alekseev (2019). The *serrulatus* group of the cyclopid freshwater genus *Eucyclops* comprises species having 12-segmented antennules with the three most distal segments possessing a smooth hyaline membrane, caudal rami 3.5-7 times as long as wide with a longitudinal row of spinules along most of the outer edge of each ramus, a strong inner spine on P5 and a number of microcharacters presented on the antennary basipodite and the caudal surface of P4 coxopodite, as described by Alekseev and Defaye (2011). The type species of this genus is *Eucyclopsserrulatus* (Fischer, 1851). This species has been reported from waterbodies around the world (Dussart and Defaye 1985, Dussart and Defaye 2006) and, consequently, it has long been considered a cosmopolitan species until some species that look like *Eucyclopsserrulatus* were separated from it (Dussart 1984, Reid 1995, Ishida 1997, Ishida 1998). Some of these may indeed be valid species; others were forms of *Eucyclopsserrulatus*. Considerable interpopulation variability in *E.serrulatus* was described; some of them are cryptic taxa and need to be revised (Sukhikh and Alekseev 2015). *Eucyclopsserrulatus* has been observed in the samples collected in Algeria in the Hoggar, south of Tassili n’Ajjer, in Tamanrasset and in Guelma (Roy and Gauthier 1927). The re-description of the type for *Eucyclopsserrulatus* (Alekseev et al. 2006) using molecular-techniques revealed several microcharacters, important for the species identification as ornamentation of antennary (A2) basipodite and the fourth leg (P4) coxopodite with coxal spine. The name ‘*serrulatus* group’ is used for the *E.serrulatus* species complex and it was established by Kiefer in 1928 for a group of tropical species closely related to *E.serrulatus* (Alekseev and Defaye 2011). The *serrulatus* group now includes 17 species and subspecies, which differ from each other in the presence of microcharacters of the antennary basipodite and the P4 coxopodite. The aim of this work is to describe and compare Algerian morphotypes of *Eucyclopsserrulatus* group coming from different localities.

## Materials and methods

**Figure 1.**
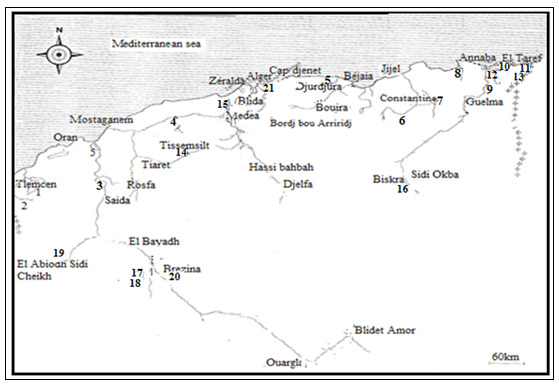
Mapping of sampling localities (numbers referred to localities) for morphotypes of Eucyclopscf.serrulatus (Fischer, 1851). Morphotype 1: (1-15); morphotype 2: (16-20); morphotype 3: (21).

Several water bodies were sampled in different regions in Algeria. Eucyclopscf.serrulatus (Fischer, 1851) was found in 21 localities (Fig. 1, Table 1). All samples were collected by horizontal trawl at 1 m depth in the near-shore areas using standard plankton net of 50-μm mesh size. Samples were collected by the author. The samples were preserved in 70% ethanol. For the measurements, six females of each population collected from the field were first placed in small Petri dish containing a mixture of alcohol, water and glycerine. Once the water evaporated (1-2 days), body length, relative length of antennules, the fourth and fifth legs and caudal rami were measured (Table 2). The specimens were dissected in concentrated glycerine and put between slide and coverslip in a drop of glycerol. The measurements and the drawings were made using a drawing tube attached to the microscope. The morphotypes were described, based on the habitus and the microcharacters: the ornamentation of antennule (A1) and antennary (A2) basipodite, caudal surface ornamentation of P4 coxopodite and intercoxal plate, using the coding system for microcharacters in *Eucyclops* proposed by Alekseev et al. (2006).

## Taxon treatments

### Eucyclops serrulatus

Fisher, 1851

40DDC8A1-F443-590D-8EA4-BB25BE963F8D

Synonymy in Dussart and Defaye (1985)
Cyclope serrulatus , Fischer, 1851
Eucyclops serrulatus , Claus, 1893a
Cyclope agilis , Gurney, 1933
Eucyclops agilis , Comita, 1951
Eucyclops serrulatus , Dussart, 1969; Kiefer, 1978
Synonymy in Dumont (1979)
Eucyclops asymmetricus , Dumont and Pensaert, 1979

#### Materials

- **Type status:** Other material. **Occurrence:** sex: Females; occurrenceID: 426B0CAF-F32A-51D1-ACE2-BA77EFA869A6; **Taxon:** scientificName: Eucyclopscf.serrulatus; class: Copepoda; family: Cyclopidae; genus: Eucyclops; scientificNameAuthorship: Fisher, 1851; **Location:** higherGeography: North Africa; continent: Africa; waterBody: Freshwater; country: Algeria; countryCode: Algeria/DZ; locality: 1-Terni wadi (Tlemcen); verbatimElevation: 867 m; verbatimCoordinates: 34°47'45"N 01°21'32"W
- **Type status:** Other material. **Occurrence:** sex: Males, Females; occurrenceID: 442B1B63-FB26-5124-99CF-685A20325890; **Taxon:** scientificName: Eucyclopscf.serrulatus; class: Copepoda; family: Cyclopidae; genus: Eucyclops; scientificNameAuthorship: Fisher, 1851; **Location:** higherGeography: North Africa; continent: Africa; waterBody: Freshwater; country: Algeria; countryCode: Algeria/DZ; locality: 2.Tafna source(Tlemcen); verbatimElevation: 867 m; verbatimCoordinates: 34°39′48″N 01°20′02″W
- **Type status:** Other material. **Occurrence:** sex: Females; occurrenceID: 31CE7192-D47F-54C0-8366-D22EFED3264F; **Taxon:** scientificName: Eucyclopscf.serrulatus; class: Copepoda; family: Cyclopidae; genus: Eucyclops; scientificNameAuthorship: Fisher, 1851; **Location:** higherGeography: North Africa; continent: Africa; waterBody: Freshwater; country: Algeria; countryCode: Algeria/DZ; locality: 3. Saida wadi (Saida); verbatimElevation: 980 m; verbatimCoordinates: 34°55′0″N, 0°13′0″W
- **Type status:** Other material. **Occurrence:** sex: Males, Females; occurrenceID: 32E56668-AD2A-58A7-8C78-5BE0D8305EAA; **Taxon:** scientificName: Eucyclopscf.serrulatus; class: Copepoda; family: Cyclopidae; genus: Eucyclops; scientificNameAuthorship: Fisher, 1851; **Location:** higherGeography: North Africa; continent: Africa; waterBody: Freshwater; country: Algeria; countryCode: Algeria/DZ; locality: 4.Chellif wadi (Ech Chellif); verbatimElevation: 86 m; verbatimCoordinates: 36°02′22″N 0°07′55″E
- **Type status:** Other material. **Occurrence:** sex: Males, Females; occurrenceID: 3EAF7316-D209-5E3D-829B-27F274092B92; **Taxon:** scientificName: Eucyclopscf.serrulatus; class: Copepoda; family: Cyclopidae; genus: Eucyclops; scientificNameAuthorship: Fisher, 1851; **Location:** higherGeography: North Africa; continent: Africa; waterBody: Freshwater; country: Algeria; countryCode: Algeria/DZ; locality: 5. Basins of Djurdjura (Bouira); verbatimElevation: 2308 m; verbatimCoordinates: 28°00N 03°00E
- **Type status:** Other material. **Occurrence:** sex: Females; occurrenceID: 890E0C23-240C-5743-BE15-017B39EDE754; **Taxon:** scientificName: Eucyclopscf.serrulatus; class: Copepoda; family: Cyclopidae; genus: Eucyclops; scientificNameAuthorship: Fisher, 1851; **Location:** higherGeography: North Africa; continent: Africa; waterBody: Freshwater; country: Algeria; countryCode: Algeria/DZ; locality: 6.Rhumel wadi (Constantine); verbatimElevation: 1090 m; verbatimCoordinates: 36°32'17"N 1°15'59"E
- **Type status:** Other material. **Occurrence:** sex: Males, Females; occurrenceID: 1080AA15-4DAB-5C27-8C38-0D9A25A48EF2; **Taxon:** scientificName: Eucyclopscf.serrulatus; class: Copepoda; family: Cyclopidae; genus: Eucyclops; scientificNameAuthorship: Fisher, 1851; **Location:** higherGeography: North Africa; continent: Africa; waterBody: Freshwater; country: Algeria; countryCode: Algeria/DZ; locality: 7. Boumerzoug wadi (Constantine); verbatimElevation: 506 m; verbatimCoordinates: 36°21'3"N 06°37'2"E
- **Type status:** Other material. **Occurrence:** sex: Males, Females; occurrenceID: 5D2B1234-2603-5E8E-B3D7-FB0C4D729FF9; **Taxon:** scientificName: Eucyclopscf.serrulatus; class: Copepoda; family: Cyclopidae; genus: Eucyclops; scientificNameAuthorship: Fisher, 1851; **Location:** higherGeography: North Africa; continent: Africa; waterBody: Freshwater; country: Algeria; countryCode: Algeria/DZ; locality: 8. Benazouz wadi (Skikda); verbatimElevation: 17 m; verbatimCoordinates: 35°27'0"N 03°51'0"E
- **Type status:** Other material. **Occurrence:** sex: Males, Females; occurrenceID: 22BE37AC-5089-530A-9ADC-CCE8EC01C51A; **Taxon:** scientificName: Eucyclopscf.serrulatus; class: Copepoda; family: Cyclopidae; genus: Eucyclops; scientificNameAuthorship: Fisher, 1851; **Location:** higherGeography: North Africa; continent: Africa; waterBody: Freshwater; country: Algeria; countryCode: Algeria/DZ; locality: 9. Seybouse wadi (Annaba); verbatimElevation: 0 m; verbatimCoordinates: 36°52′01″N 07°46′18″E
- **Type status:** Other material. **Occurrence:** sex: Males, Females; occurrenceID: 481FB5C6-96E0-57A0-97DC-660499B898B6; **Taxon:** scientificName: Eucyclopscf.serrulatus; class: Copepoda; family: Cyclopidae; genus: Eucyclops; scientificNameAuthorship: Fisher, 1851; **Location:** higherGeography: North Africa; continent: Africa; waterBody: Freshwater; country: Algeria; countryCode: Algeria/DZ; locality: 10. Lake of Oubeira (El Taref); verbatimElevation: 25 m; verbatimCoordinates: 36°50'695 N 8°23'272 E
- **Type status:** Other material. **Occurrence:** sex: Males, Females; occurrenceID: 2D38EC65-8DF5-5AEB-A6E7-7CD09C57A3F1; **Taxon:** scientificName: Eucyclopscf.serrulatus; class: Copepoda; family: Cyclopidae; genus: Eucyclops; scientificNameAuthorship: Fisher, 1851; **Location:** higherGeography: North Africa; continent: Africa; waterBody: Freshwater; country: Algeria; countryCode: Algeria/DZ; locality: 11. Lake of Tonga; verbatimElevation: 589-1061 m; verbatimCoordinates: 36°51'511 N 8°30’100 E
- **Type status:** Other material. **Occurrence:** sex: Males, Females; occurrenceID: 251DAD60-F172-5E97-AFA6-CE64E09174E8; **Taxon:** scientificName: Eucyclopscf.serrulatus; class: Copepoda; family: Cyclopidae; genus: Eucyclops; scientificNameAuthorship: Fisher, 1851; **Location:** higherGeography: North Africa; continent: Africa; waterBody: Freshwater; country: Algeria; countryCode: Algeria/DZ; locality: 12. Blue lake (El Taref); verbatimElevation: 1-123 m; verbatimCoordinates: 36°31'60" N 07°40'0" E
- **Type status:** Other material. **Occurrence:** sex: Males, Females; occurrenceID: CBF843B7-5FB2-550F-9AB7-B9FAA949BDB1; **Taxon:** scientificName: Eucyclopscf.serrulatus; class: Copepoda; family: Cyclopidae; genus: Eucyclops; scientificNameAuthorship: Fisher, 1851; **Location:** higherGeography: North Africa; continent: Africa; waterBody: Freshwater; country: Algeria; countryCode: Algeria/DZ; locality: 13. Messida wadi; verbatimElevation: 1 m; verbatimCoordinates: 36°54'0" N 08°31'0" E
- **Type status:** Other material. **Occurrence:** sex: Males, Females; occurrenceID: 6616E321-7046-5FC0-B0DC-FE94C8D3CB7A; **Taxon:** scientificName: Eucyclopscf.serrulatus; class: Copepoda; family: Cyclopidae; genus: Eucyclops; scientificNameAuthorship: Fisher, 1851; **Location:** higherGeography: North Africa; continent: Africa; waterBody: Freshwater; country: Algeria; countryCode: Algeria/DZ; locality: 14. Basins (Tasslemt,Tissemssilt); verbatimElevation: 900 m; verbatimCoordinates: 35°36′00,00″ N 1°49′00,00″ E
- **Type status:** Other material. **Occurrence:** sex: Males, Females; occurrenceID: F9C0C57B-BCEC-519D-9941-E3A2B25962F3; **Taxon:** scientificName: Eucyclopscf.serrulatus; class: Copepoda; family: Cyclopidae; genus: Eucyclops; scientificNameAuthorship: Fisher, 1851; **Location:** higherGeography: North Africa; continent: Africa; waterBody: Freshwater; country: Algeria; countryCode: Algeria/DZ; locality: 15. Basins (Tamezguida,Medea)”; verbatimElevation: 591 m; verbatimCoordinates: 36°19′27″ N 02°41′22″ E
- **Type status:** Other material. **Occurrence:** sex: Males, Females; occurrenceID: 584E7A11-5DB1-5277-BDD9-F255530B0F53; **Taxon:** scientificName: Eucyclopscf.serrulatus; class: Copepoda; family: Cyclopidae; genus: Eucyclops; scientificNameAuthorship: Fisher, 1851; **Location:** higherGeography: North Africa; continent: Africa; waterBody: Freshwater; country: Algeria; countryCode: Algeria/DZ; locality: 16.Seggerwadi(Biskra); verbatimElevation: 87 m; verbatimCoordinates: 34°0′0″N 5°0′0″E
- **Type status:** Other material. **Occurrence:** sex: Males, Females; occurrenceID: B4824273-01D1-5C1D-85A2-41A11DDE507F; **Taxon:** scientificName: Eucyclopscf.serrulatus; class: Copepoda; family: Cyclopidae; genus: Eucyclops; scientificNameAuthorship: Fisher, 1851; **Location:** higherGeography: North Africa; continent: Africa; waterBody: Freshwater; country: Algeria; countryCode: Algeria/DZ; locality: 17. Lake of Ain Saadane (El Biodh Sidi Cheich); verbatimElevation: 744 m; verbatimCoordinates: 32°53′55″ N 0°32′22″ E
- **Type status:** Other material. **Occurrence:** sex: Males, Females; occurrenceID: A26C75B4-6981-5E00-BAB5-6B30B87D624B; **Taxon:** scientificName: Eucyclopscf.serrulatus; class: Copepoda; family: Cyclopidae; genus: Eucyclops; scientificNameAuthorship: Fisher, 1851; **Location:** higherGeography: North Africa; continent: Africa; waterBody: Freshwater; country: Algeria; countryCode: Algeria/DZ; locality: 18. Source of Ain EL Hammam (Brezina-); verbatimElevation: 849 m; verbatimCoordinates: 33°05′58″ N 1°15′39″ E
- **Type status:** Other material. **Occurrence:** sex: Males, Females; occurrenceID: 46698A17-EB7C-5612-AB1C-205B7AC50B79; **Taxon:** scientificName: Eucyclopscf.serrulatus; class: Copepoda; family: Cyclopidae; genus: Eucyclops; scientificNameAuthorship: Fisher, 1851; **Location:** higherGeography: North Africa; continent: Africa; waterBody: Freshwater; country: Algeria; countryCode: Algeria/DZ; locality: 19. Lake of Gue of Arsaouet (El Biodh Sidi Cheich); verbatimElevation: 744 m; verbatimCoordinates: 32°53′55″ N 0°32′22″ E
- **Type status:** Other material. **Occurrence:** sex: Males, Females; occurrenceID: 3486980E-0645-5530-9F9D-48E53FED8E68; **Taxon:** scientificName: Eucyclopscf.serrulatus; class: Copepoda; family: Cyclopidae; genus: Eucyclops; scientificNameAuthorship: Fisher, 1851; **Location:** higherGeography: North Africa; continent: Africa; waterBody: Freshwater; country: Algeria; countryCode: Algeria/DZ; locality: 20. Source of El Goleita (Brizina); verbatimElevation: 849 m; verbatimCoordinates: 33°05′58″ N 01°15′39″ E
- **Type status:** Other material. **Occurrence:** sex: Males, Females; occurrenceID: A653531E-391F-5513-B8FF-94E1506DF9EA; **Taxon:** scientificName: Eucyclopscf.serrulatus; class: Copepoda; family: Cyclopidae; genus: Eucyclops; scientificNameAuthorship: Fisher, 1851; **Location:** higherGeography: North Africa; continent: Africa; waterBody: Freshwater; country: Algeria; countryCode: Algeria/DZ; locality: 21. Swamp (El-Harrach, Algiers); verbatimElevation: 0-178 m; verbatimCoordinates: 36°43′16″ N 03°08′15″ E

#### Description

**Figure 2.**
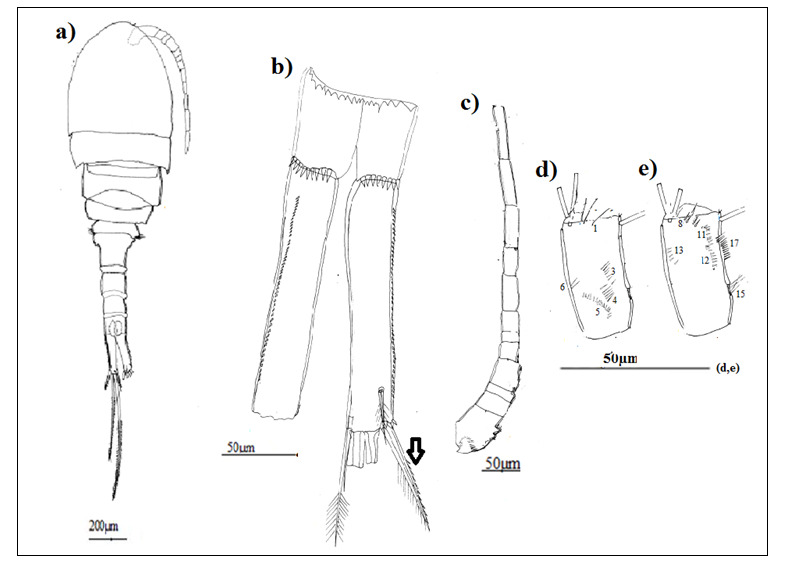
Eucyclopscf.serrulatus (Fischer, 1851). Morphotype 1; **a** Habitus (dorsal view); **b** caudal rami; **c** A1; **d** A2 basipodite (posterior surface); **e** A2 basipodite (anterior surface).

Based on habitus, morphotype 3 of *Eucyclopsserrulatus* group was the smallest one (Table 2), morphotype 1 is characterised by external articulation of its abdominal segments (Fig. 2a), while morphotype 2 is identified by its elongated lateral thoracic segments which envelop the following segment and its fourth thoracic segment has cilia on its lateral angles (Fig. 4a).

**Figure 3.**
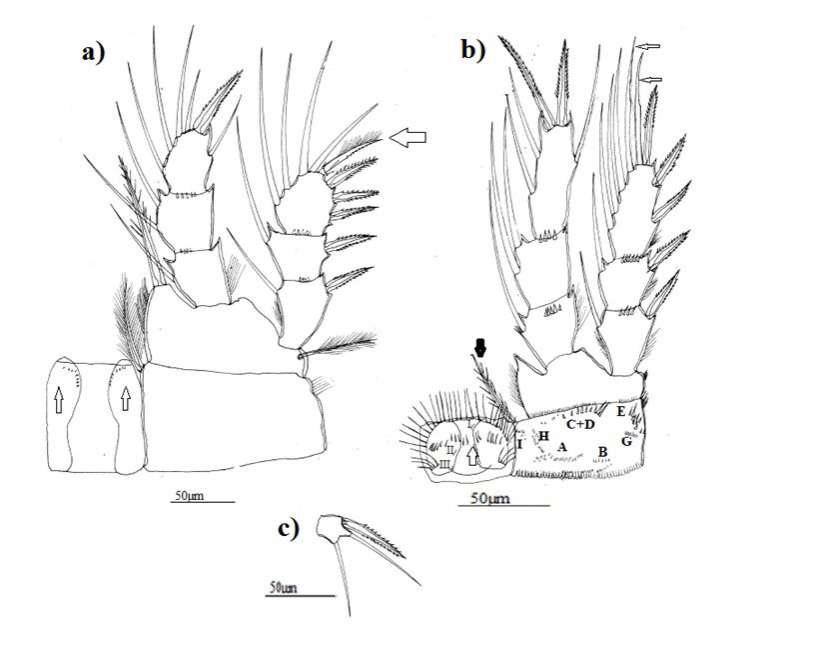
Eucyclopscf.serrulatus (Fischer, 1851). Morphotype 1 **a** first leg (P1) with coxopodite and intercoxal plate (with fine denticules), arrow show external seta of exopodite 3 with a row of little spinules along outer margin and with setules on inner edge; **b** fourth leg (P4) with coxopodite and intercoxal plate, arrows showing two apical setae of exopodite 3 stylet-shaped; **c** fifth leg (P5).

P5 with a spine as long as outer seta in all morphotypes, slender and long in morphotypes 1 and 3 (Fig. 3c, Fig. 7c) and large in morphotype 2 (Fig. 5c).

**Figure 4.**
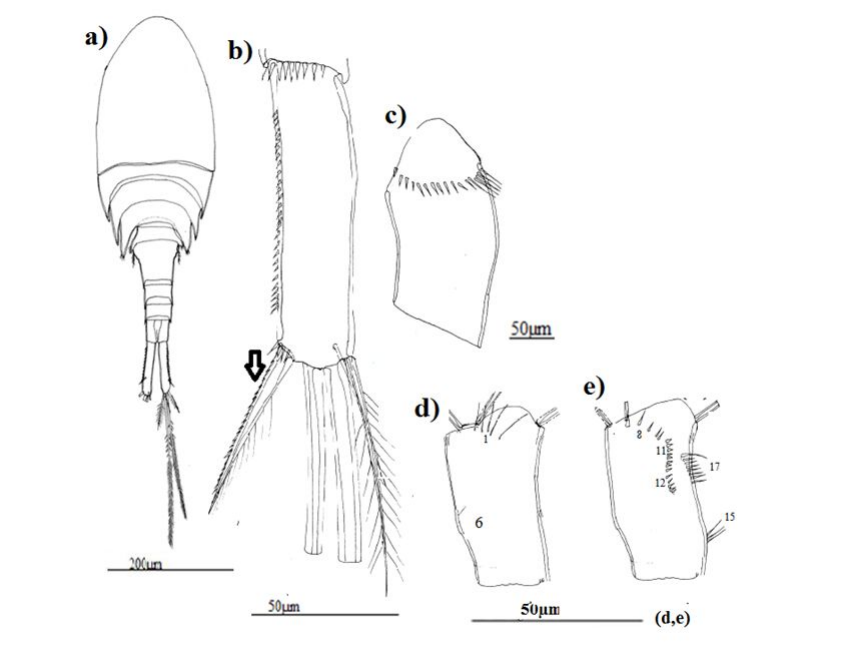
Eucyclopscf.serrulatus (Fischer, 1851). Morphotype 2; **a** habitus (dorsal view); **b** caudal rami; **c** A1 basipodite; **d** A2 basipodite (posterior surface); **e** A2 basipodite (anterior surface).

Caudal rami somewhat divergent: morphotype 1:4–4.5 times longer than wide (Fig. 2b), morphotype 2:4.5–5 times longer than wide (Fig. 4b), morphotype 3:3.5–4 times longer than wide (Fig. 6b). Serra (longitudinal row of spinules) with 51 denticules in morphotype 1, 28 denticules in morphotype 2 and 22 denticules in morphotype 3, spine-like outermost seta with spinules along outer margin and long setules on inner edge in all the morphotypes. Innermost seta with long setules on both sides, about 1.3–1.4 times longer than spine-like outermost seta in morphotypes 2 and 3, but almost equal in morphotype 1.

Antennule 12-segmented, reaching middle of first free thoracal somite in morphotypes 1 and 2 and the beginning of the third one in morphotype 3 (Fig. 2a, Fig. 6a), the last three articles with hyaline membrane, the first segment with curved row of spinules at its base; outermost spinules the longest in morphotypes 1 and 2 (Fig. 2c, Fig. 4c). In morphotype 3, the longest ones were between two groups of little spines (Fig. 6c).

Antennary basipodite, posterior face: (N1) with three long setules in morphotypes 1 and 3 (Fig. 2d, Fig. 6d), five long setules in morphotype 2 (Fig. 4d); a group of two long spinules (N6) and three diagonal and parallel rows of spinules (N3–5) only in morphotype 1.

Antennary basipodite, anterior face: (N8) composed of three long spinules subdistally in morphotype 1 (Fig. 2e), five long spinules subdistally in morphotype 2 and three (Fig. 4e, Fig. 6e), (N11 + N12) formed of a long row of relatively small spinules with 17 spinules in morphotype 1, 13 spinules in morphotype 2. In morphotype 3 only (N11) exist with eight spinules, (N13) represented by a group of five little spinules only in morphotype 1, two groups of marginal spinules (N17) and (N15) only in morphotypes 1 and 2, in morphotype 3 only (N17) exist.

Endopodite and exopodite segments of P1–P4 were plumose.

**Figure 5.**
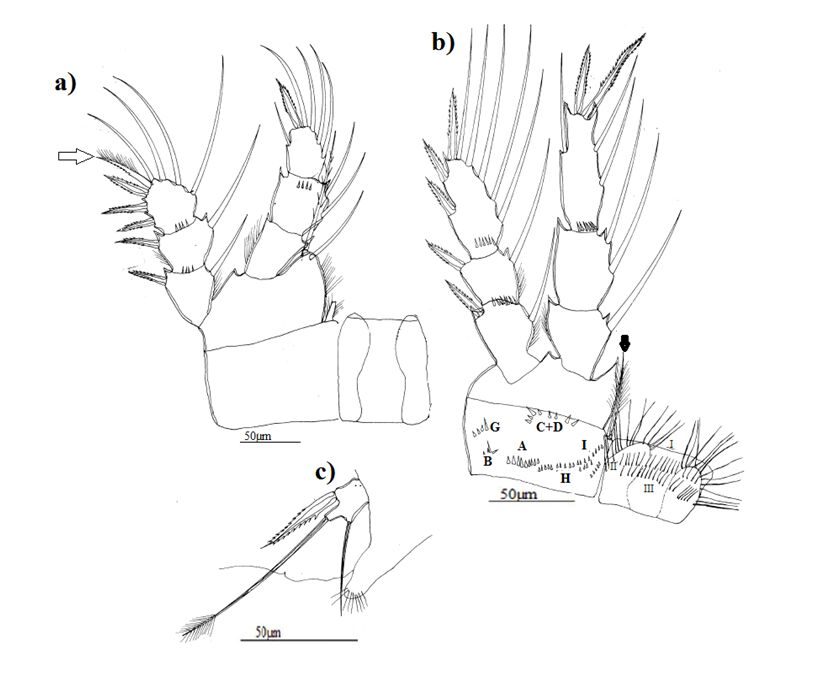
Eucyclopscf.serrulatus (Fischer, 1851). Morphotype 2; **a** first leg (P1) with coxopodite and intercoxal plate, arrow showing external seta of exopodite 3 with a row of little spinules along outer margin and with setules on inner edge; **b** fourth leg (P4) with coxopodite and intercoxal plate; **c** fifth leg (P5).

**Figure 6.**
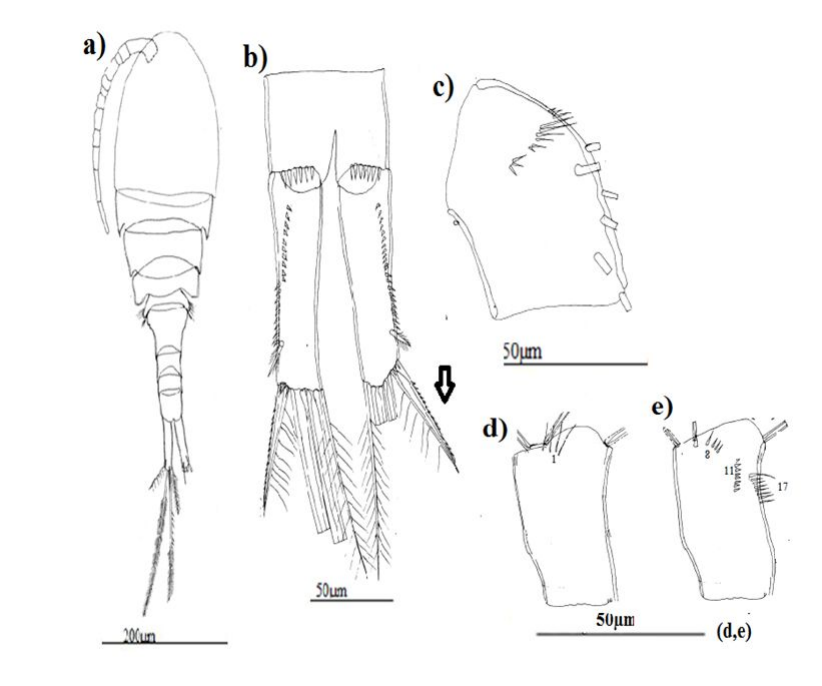
Eucyclopscf.serrulatus (Fischer, 1851). Morphotype 3; **a** habitus (dorsal view); **b** caudal rami; **c** A1 basipodite; **d** A2 basipodite (posterior surface); **e** A2 basipodite (anterior surface).

**Figure 7.**
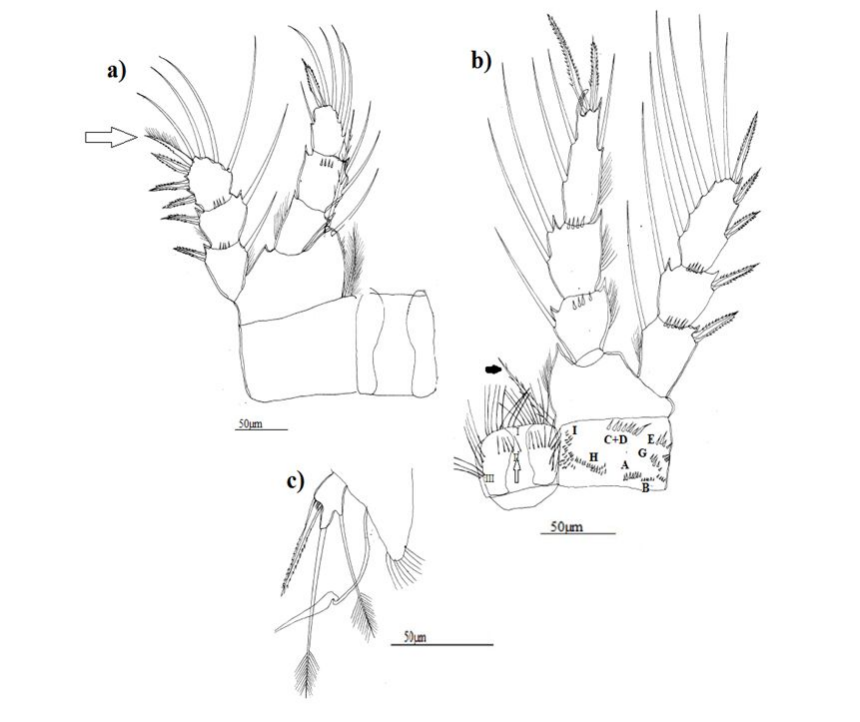
Eucyclopscf.serrulatus (Fischer, 1851). Morphotype 3; **a** first leg (P1) with coxopodite and intercoxal plate, arrow showing external seta of exopodite 3 with a row of little spinules along outer margin and with setules on inner edge; **b** fourth leg (P4) with coxopodite and intercoxal plate; **c** fifth leg (P5).

P1: inner edge of basipodite with group of long hair only in morphotypes 1 and 2 (Fig. 3a, Fig. 5a), intercoxal plate with two groups of finest spinules on body of protuberances, only in morphotype 1, external seta of exopodite 3 in all morphotypes with a row of little spinules along outer margin and with long setules on inner edge (Fig. 3a, Fig. 5a, Fig. 7a).

P4, innermost apical spine of endopodite 3 was 1.3–1.4 times as long as outermost apical spine in all morphotypes and about 1.5-1.6 times as long as supporting segment in morphotype 1 (Fig. 3b) and as long as supporting segment in morphotypes 2 and 3 (Fig. 5b, Fig. 7b), outer seta long reaching almost the top of outermost apical spine in all morphotypes, two apical setae of exopodite 3 stylet-shaped only in morphotype 1 (Fig. 3b), inner edge of basipodite with group of long setules only in morphotypes 1 and 3 (Fig. 3b, Fig. 7b). Coxopodite with a row of numerous fines spinules along internal distal side only in morphotype 1, several groups of spinules: (A – B - (C + D) –E-G-H-I) in morphotype 1, (A – B- (C + D) – G - H –I) in morphotype 2 and (A – B- (C + D) – E- G- H- I) in morphotype 3, morphotype 2 did not show group E. Group F did not exist in all morphotypes, intercoxal plate with dense setules, in all morphotypes, but in morphotypes 2 and 3, setules are two times longer than those in morphotype 1. On body of plate, I found two groups of little setules and spinules in morphotype 1, two groups of little spinules in morphotype 2 and one group of little setules in morphotype 3. Caudal setae had dense long setules, but those of morphotypes 1 and 3 had a strong spine.

#### Distribution

This taxon was discovered in the mid-nineteenth century in a pond at Peterhof close to Saint Petersburg, Russia (Fischer 1851). In recent years, the distributional area of the species was revised and restricted to a Palearctic distribution. Previous data on geographical distribution of the species outside this area are critically analysed. It is hypothesised that records of *E.serrulatus* from Japan, Australia, North America and other zoogeographical zones could be a result of recent invasions, possibly via human activities in relation to ship transport.

#### Ecology

Freshwater species (lakes, basins, ponds, wadis)

#### Taxon discussion

*Eucyclopsserrulatus* (Fischer, 1851) has been reported from waterbodies around the world and, consequently, it has long been considered a cosmopolitan species until some species that look like *Eucyclopsserrulatus* were separated from it. Some of these may indeed be valid species; others were forms of *Eucyclopsserrulatus*.

## Discussion

In Algeria, I identified three morphotypes belonging to *Eucyclopsserrulatus* group that differ from the description of *Eucyclopsserrulatus* from the type locality in the microcharacters of the antennary basipodite and of the P4 coxopodite. The characters of the most widespread morphotype (morphotype 1) were stable in all localities despite the fact that these were located in two geographically separated regions (eastern and western Algeria)

In twenty-one localities, I did not find *Eucyclopsserrulatus*, while this species has already been mentioned in Algeria by Roy and Gauthier (1927).

In a comparison with other species of *Eucyclopsserrulatus* group from North Africa, I considered *Eucyclopsserrulatushadjebensis* (Kiefer, 1926), but this latter is characterised by a shorter serra.

*Eucyclopsagiloides* (Sar, 1909) was recorded in Algeria by Defaye et al. (2010). These morphotypes (at least for morphotypes 2 and 3) cannot be attributed to this species, due to the lack of long hair-like spinules in position 1-2 on the posterior surface of antennary basipod; also the "serra" on caudal rami is completely different. P4 intercoxal plate bears long hair-like spinules in these morphotypes, but it is short in *Eucyclopsagiloides*. There was ornamentation of P4 coxopodite with 20-21 spinules (C+D) in this species, but with eight spinules in the morphotypes.

These morphotypes did not cohabit and presented important differences in morphological characters and microcharacters; they could be ascribed to new pseudocryptic species of the *Eucyclopsserrulatus* group, which is in need of urgent revision.

## Supplementary Material

XML Treatment for Eucyclops serrulatus

## Figures and Tables

**Table 1.** Occurrences of Eucyclopscf.serrulatus morphotypes in different localities (numbers referred to the localities).

Eucyclopscf.serrulatus	Localities with occurrences of the three morphotypes
Morphotype 1	(1) Females; (2) Males, females; (3) Females; (4) Males, females; (5) Several males, several females; (6) Females; (7) Males, females; (8) Males, females; (9) Females; (10) Males, females; (11) Males, females; (12) Males, females; (13) Females; (14) Several males, several females; (15) Males, females.
Morphotype 2	(16) Several male: Several females; (17) Several males, several females; (18) Several males, several females; (19) Several males, several females; (20) Several males, several females.
Morphotype 3	(21) Males, females.

**Table 2.** Female morphometric characters in morphotypes of Eucyclopscf.serrulatus.

Eucyclopscf.serrulatus	Morphotype 1		Morphotype 2		Morphotype 3	
	range	mean (mm)	range	mean (mm)	range	mean (mm)
Body length (mm)	0.86 -1	0.93	0.81 - 1	0.93	0.79 -1	0.85
Cephalothorax, length / width	1-1.2	1.08	1-1.2	1.06	1-1.18	1.05
P5, length of outer seta / length of spine	0.98-1	0.97	0.98-1	0.97	0.97-1.2	1.05
Caudal rami, length / width	4-4.4	4.2	4.5-5	4.76	3.5-4.2	3.96
Caudal rami, length of innermost setae/length of outermost spine like seta	0.8-1	0.93	1.3-1.6	1.46	1.4-1.5	1.48
P4 Enp3, length / width	2.5-3	2.8	2.3-2.9	2.6	2.4-2.7	2.6
P4 Enp3, inner apical spine /outer apical spine	1.3-1.5	1.36	1.3-1.4	1.33	1.3-1.4	1.38
P4 Enp3, inner apicalspine / segment length	1.5-1.6	1.55	0.98-1	0.96	0.99-1	0.98
Genital segment: length / width	1.1-1.2	1.15	1.1-1.3	1.14	1-1.1	0.95
